# Red Blood Cells as Endogenous Biotweezers for Optical Micromanipulation In Vivo

**DOI:** 10.1002/advs.76794

**Published:** 2026-07-23

**Authors:** Tong Yang, Xinyu Ren, Dalin Ma, Hao Pang, Wei Chen, Kaize Cai, Mei Yuan, Bingzhi Zhang, Zufang Lin, Xiaoshuai Liu

**Affiliations:** ^1^ Department of Optoelectronic Engineering School of Physics and Materials Science Guangzhou University Guangzhou Guangdong China; ^2^ College of Artificial Intelligence and Low‐Altitude Technology South China Agricultural University Guangzhou Guangdong China

**Keywords:** biophotonics, biotweezer, in vivo, optical manipulation, red blood cell

## Abstract

Precise control of micro‐/nano‐scale objects in vivo is crucial for biomedical applications. Here, we demonstrate a biohybrid optical manipulation strategy that integrates a long‐distance manipulation fiber probe (LDMFP) and natural red blood cells (RBCs) to construct endogenous biotweezers for optical micromanipulation in vivo. By using the engineered LDMFP, an extended focal field was generated, which could capture flowing RBCs within the living blood vessel. Benefiting from their smooth disk‐shape and homogenous refractive index distribution, these trapped RBCs can function as natural biotweezers that refocused incident light to create a secondary optical trap, thus achieving an enhanced microparticle trapping probability (98% vs. 5%) and improved counter‐flow migration velocity (12 vs. 4.5 µm/s) when compared to the LDMFP alone, as well as rapid immune cell activation within 10 s. By assembling multiple RBCs into extended biotweezer arrays, they can further function as biological waveguides for long‐distance directional transmission, achieving strong focusing and localized field enhancement with extended manipulation distances. Experimental results were supported by finite‐element simulations, which validated the improved trapping stiffness and operational range. This bio‐hybrid approach presents a promising platform for intravital optical manipulation and suggests potential applications in therapeutic delivery and behavior modulation of immune cells.

## Graphical abstract

By synergistically integrating the long‐distance manipulation fiber probe (LDMFP) and endogenous red blood cells (RBCs) as biocompatible optical elements, a natural RBC biotweezer was developed for the desired microparticle manipulation and immune cell activation within living vasculature, which might offer an alternative tool for intravital optical manipulation with enhanced trapping stiffness.

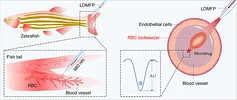

## 1 Introduction

Precise spatiotemporal control over therapeutic agents and immune cells within the living organism is crucial for advancing targeted drug delivery and immunomodulation strategies [1, 2, 3, 4, 5]. While nanoparticle‐based drug delivery and immunomodulatory approaches have shown great promise [6, 7, 8], their efficacy is often limited by the inability to actively guide these entities to specific disease sites after systemic administration [9, 10, 11], leading to suboptimal biodistribution and potential off‐target effects [12, 13, 14]. Therefore, developing a novel technology capable of non‐contact and real‐time manipulation of micro‐ and nano‐scale targets in vivo would represent a fascinating tool for both fundamental research and clinical applications [15]. Established physical manipulation techniques, such as magnetic tweezers [16, 17], acoustic tweezers [18, 19], and microfluidic‐based methods [20, 21], have demonstrated remarkable capabilities for intravital micromanipulation, although they often involve extra functional modifications or limited operational precision [22]. Optical manipulation, leveraging its non‐invasive and high‐precision nature, has emerged as a promising alternative [23, 24]. However, conventional optical tweezers are constrained by the bulky structure and limited operation flexibility of high‐numerical‐aperture objectives [25], preventing their use in complex vascular networks [26, 27]. While optical fiber tweezers offer improved flexibility [28, 29], most existing probes typically generate a strong gradient force only in close proximity to the fiber tip [30, 31]. This is inadequate for in vivo applications, where targets are shielded by vascular walls and epidermal tissues and are subject to rapid blood flow.

Recent studies have suggested that red blood cells (RBCs) can function as a biological optical element [32, 33], exhibiting light waveguiding and focusing capabilities that could potentially guide and concentrate light in vitro [34]. This intrinsic property suggests that RBCs themselves could be engineered in situ as biocompatible optical tools to extend the focus depth of the light beam [35, 36]. Inspired by this, we performed a proof‐of‐concept study to assemble an endogenous RBC biotweezer for the desired intravital micromanipulation, which was achieved by an organic integration of a long‐distance manipulation fiber probe (LDMFP) and the intrinsic optical focusing properties of RBCs. The unique long‐distance focusing capability of the LDMFP enables its emitted laser beam to penetrate epidermal tissues and vascular walls, forming stable optical potential wells within the blood vessels and allowing for the 3D manipulation of flowing RBCs. Once trapped by the LDMFP, the RBC functions not merely as a passive target but transforms into an active biotweezer that re‐concentrates the diverging laser beam to create a secondary and highly localized optical trap at its distal surface. Through validation in a zebrafish model, we present preliminary evidence demonstrating the potential of this strategy for microparticle manipulation and immune cell activation within living vasculature. Supported by numerical simulations, this work might provide an alternative strategy for future investigations into targeted therapeutic delivery and behavior modulation of immune cells in vivo.

## 2 Results

### 2.1 Schematic Illustration and Material Characterization

Figure 1a shows the schematic illustration of the proposed RBC biotweezers in vivo. To construct RBC biotweezers, the primary step involves achieving non‐contact trapping and flexible manipulation of naturally flowing RBCs within living vasculatures. Nevertheless, this presents a significant challenge as RBCs are covered by both the vascular wall and epidermal tissues, coupled with their rapid motion through the complex vascular network. To address this, an LDMFP was carefully designed and experimentally fabricated, which enables the tight focus of the emitted laser beam to penetrate the epidermis and vascular wall and then be exerted on targeted RBCs. Consequently, this generates a sufficient optical gradient force to counterbalance the hydrodynamic viscous forces exerted by blood flow, thereby achieving a stable trapping of RBCs (inset of Figure 1a). Upon stable trapping of the target RBC, the captured cell functions as a natural biotweezers, re‐focusing the divergent laser beam at its distal end to form a robust optical potential well. This secondary focusing facilitates the flexible manipulation of microscopic targets (inset of Figure 1b), as well as enhanced activation of immune cells. Crucially, this strategy leverages endogenous RBCs as biocompatible optical components, eliminating the need for complex microfabrication processes or invasive exogenous implantation. Moreover, the integration of LDMFP circumvents the reliance on high‐numerical‐aperture objectives, which not only promotes functionalization and integration but also holds promise for extending penetration depth in vivo.

**FIGURE 1:**
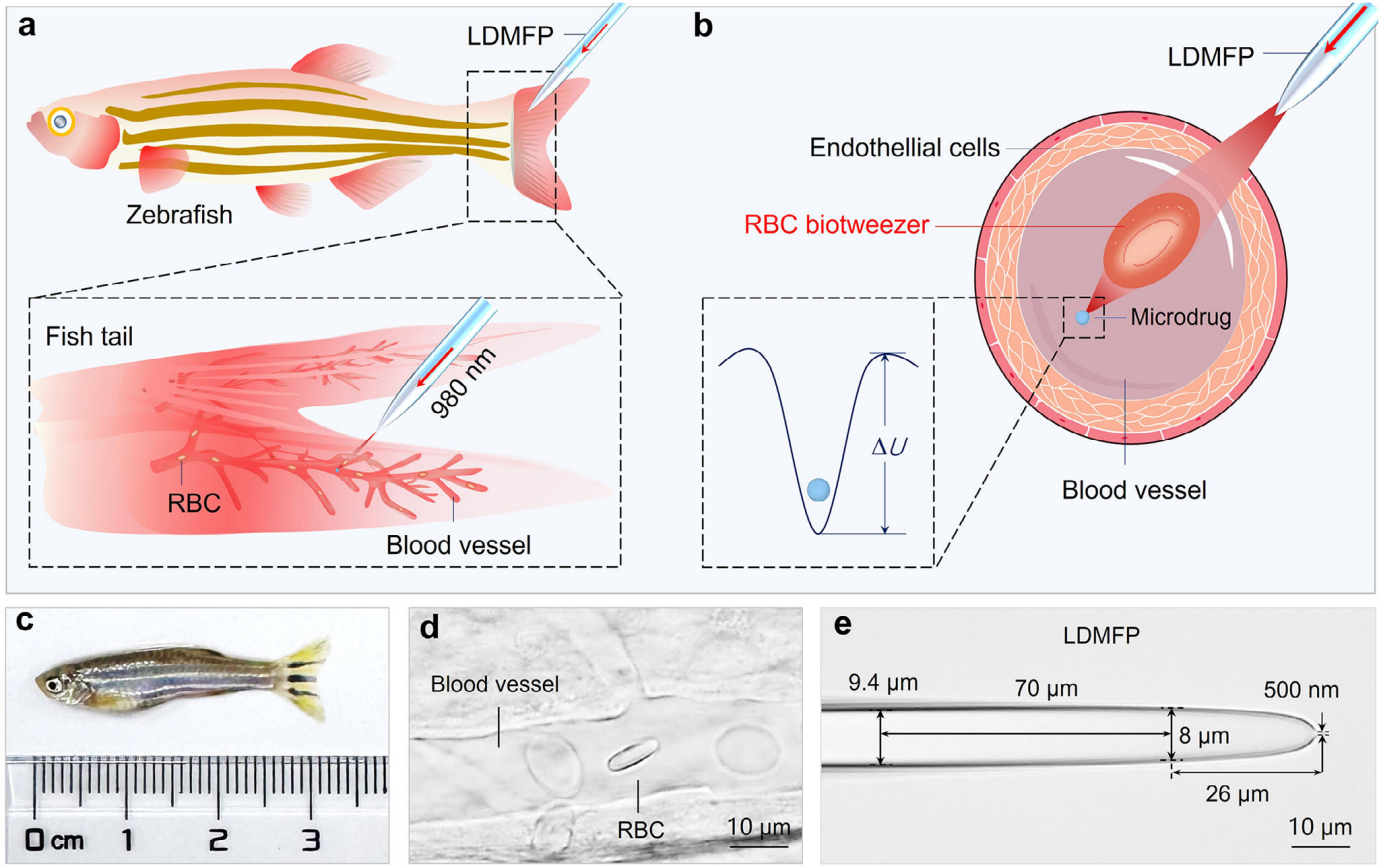
Schematic illustration and material characterization. (a) Schematic illustrating the dynamic assembly of RBC biotweezers by using LDMFP to trap RBCs in living zebrafish. The inset shows the detailed trapping process. (b) Schematic illustrating the stable trapping of an intravital microparticle by the natural RBC biotweezers. The inset shows the optical potential well formed by the RBC biotweezers with a maximum potential difference of *ΔU*. (c) Optical micrographs of adult zebrafish with an average length of 3.2 cm. (d) Optical micrographs of the naturally flowing RBCs in the blood vessel. (e) Optical micrographs of the designed LDMFP, which show a parabolic shape with a nanotip at its tapered end.

In this study, the zebrafish were selected to conduct the RBC biotweezer studies (Figure 1c), leveraging their excellent optical transparency [37, 38], high genetic homology with humans, and the availability of diverse transgenic phenotypes amenable to specific fluorescent labeling [39]. Crucially, the optical transparency of the zebrafish tail enables clear visualization of RBCs within blood vessels (Figure 1d), while allowing tissue characterization at the single‐cell level, thereby providing an ideal platform to construct endogenous RBC biotweezers in vivo. The RBCs exhibited a biconcave shape with their long axis and short axis of 10 ± 1.2 µm and 6 ± 1.2 µm, respectively (Figure S1). For the designed LDMFP, it was fabricated by a flame‐ heating technique (see materials and methods) and featured a smooth parabolic tip profile with the diameter tapering from 9.4 µm to 8 µm over a 70 µm length, followed by a further reduction from 8 µm to 500 nm within a 26 µm span (Figure 1e).

### 2.2 Optical Manipulation and Focus Characterization of RBCs In Vivo

To achieve flexible manipulation of RBCs, the LDMFP was fixed on a six‐axis microstage to achieve dynamic position modulation (resolution: 500 nm) while its end was connected to the 980‐nm laser beam, which was chosen due to its low tissue absorption and large penetration depth [40]. Meanwhile, its probe tip was modulated to approach the zebrafish tail, which was positioned onto an *x*‐*y* manual translation stage to achieve fine positioning and mechanical stability (Figure S2). All the experiments were carried out under an inverted fluorescence microscope coupled with a charge‐coupled device camera for real‐time image acquisition and video recording. Crucially, a short‐pass filter (cutoff wavelength: 700 nm) was placed in front of the CCD to prevent the manipulation laser from entering and interfering with the actual observation of the experiment process. As shown in Figure 2a1, RBCs in the blood vessel were flowing upward at a velocity of 20 ± 3 µm/s (indicated by the deep red arrow), and meanwhile, the LDMFP was positioned in the focal plane of the RBCs with its optical axis perpendicular to the vessel wall. After the 980‐nm laser launched into the probe (*P* = 70 mW), the flowing RBCs exhibited a gradual deceleration as they approached the probe tip until stable trapping was achieved. Unlike conventional fiber tweezer configurations where targets are trapped directly at the probe tip, the trapped RBC was maintained at a long distance of *d* = 30 µm from the probe end, demonstrating its long‐distance manipulation capability of naturally flowing RBCs in vivo. To confirm that observed trapping resulted from optical forces rather than stochastic adhesion, the laser beam was deactivated, upon which the target RBC immediately resumed natural flow and moved out of the field of view (Figure S3). Following stable trapping, the fiber probe was translated downward along the *y*‐axis, inducing synchronous displacement of the trapped RBC by 13 µm at *t* = 1 s while maintaining alignment between the cell center and the optical axis of LDMFP (Figure 2a2). Repetitive vertical manipulation further demonstrated reversible displacement, confirming the high operational flexibility and positional reproducibility (Figure 2a3,a4). Additionally, the adjustment of the fiber probe along the *z*‐axis enabled synergetic migration of the target cell (Figure S4), verifying the 3D manipulation capability of the proposed LDMFP.

**FIGURE 2:**
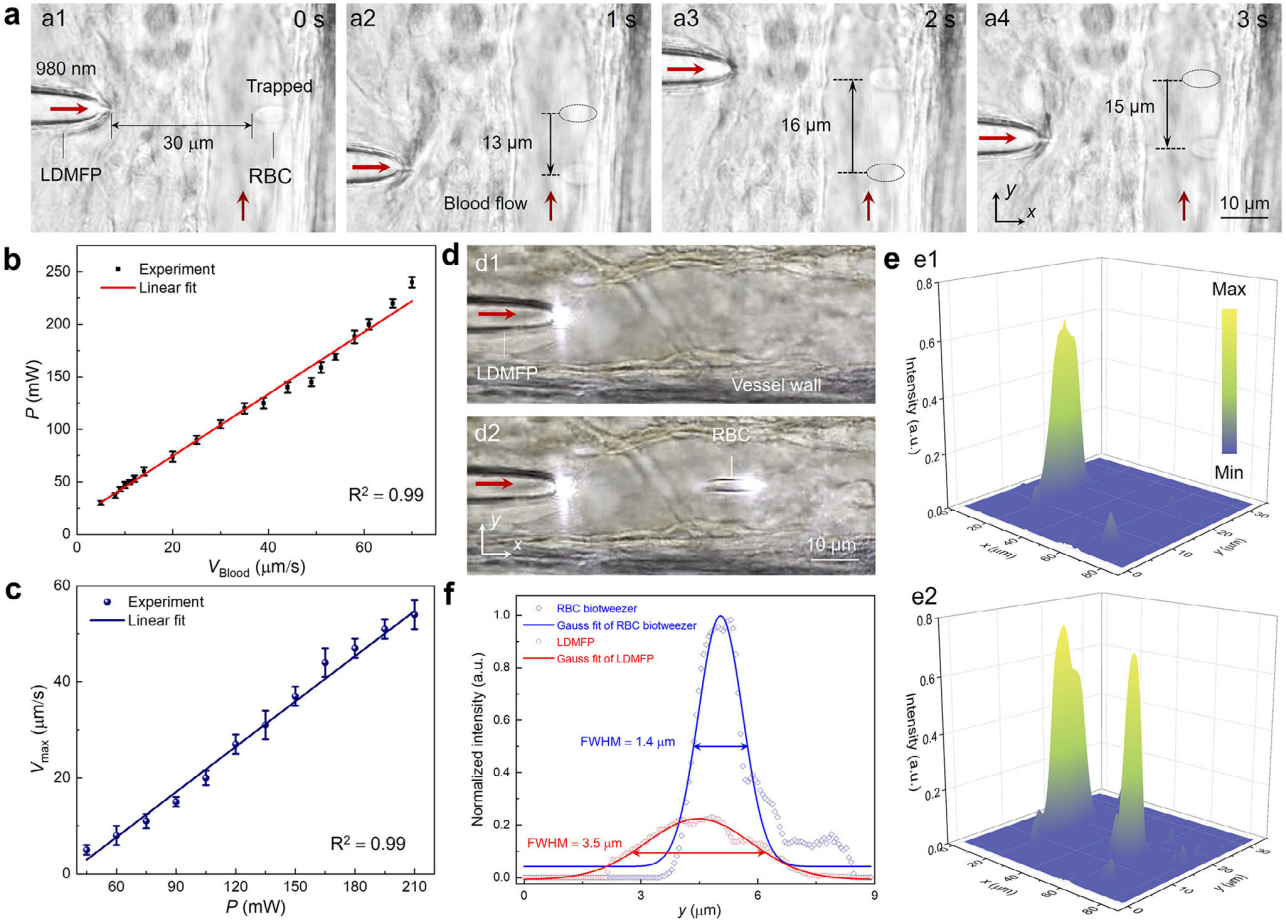
Optical manipulation and focus characterization of RBCs in vivo using the LDMFP. (a) Stable trapping (a1) and dynamic shift (a2–a4) of a single RBC in blood flow by using the LDMFP (*P* = 70 mW, *V*_blood_ = 20 ± 3 µm/s and *d* = 30 µm). (b) The required laser power for stable RBC trapping in living zebrafish as a function of blood flow velocity. Results are presented as mean ± SD (*n* = 5 individual zebrafish). (c) Maximum counter‐flow migration velocity as a function of laser power. Results are presented as mean ± SD (*n* = 5 individual zebrafish with 3 independent replicates per fish). (d) Intensity distribution of the emitted light field inside the blood vessel without (d1) and with (d2) the RBC biotweezer (*P* = 80 mW, *V*_blood_ = 24 ± 4 µm/s and *d* = 32 µm). (e) 3D color mapping of the emitted light field without (e1) and with the RBC biotweezers (e2). (f) Experimental measurement of the FWHM of the output optical field with and without the RBC biotweezer.

To demonstrate the biological compatibility of the LDMFP [41, 42], the RBC was optically trapped at different laser power levels for 60 s, with its morphological integrity and viability systematically assessed (Figure S5). Notably, the aspect ratio and morphology of RBC remain unchanged under the laser irradiation with a maximum power of 250 mW, confirming the negligible optical damage during optical manipulation by using LDMFP. In addition, the optical trapping stiffness (*k_trap_*) has been experimentally measured using the power spectral density (PSD) analysis of positional fluctuations (Figure S6). Notably, the power spectrum of the RBC fluctuation yields a Lorentzian function with a corner frequency of *f*_c_ = 19.84 Hz, from which the trapping stiffness was measured to be *k* = 212 pN/µm/W (see Supporting Information for the calculation details). Furthermore, the manipulation performance was quantitatively characterized for the LDMFP on intravital RBCs within blood flow. As the flow velocity rose from 5 ± 1 µm/s to 70 ± 6 µm/s, the required laser power (*P*) increased linearly from 30 to 240 mW. This correlation stems from the greater hydrodynamic viscous force exerted on the cell at higher flow rates, which requires a stronger optical gradient force to achieve stable trapping (Figure 2b). After achieving stable capture, the applied laser power also directly influenced the maximum migration speed of the cell. As shown in Figure 2c, under a constant blood flow velocity (*V*_blood_ = 10 ± 2 µm/s), the achievable migration speed against the flow direction increased from 5 ± 1 µm/s to 54 ± 3 µm/s as the laser power was raised from 45 to 210 mW. This is because a higher migration speed faces greater viscous resistance to overcome, necessitating a stronger optical force for stable manipulation.

Following the RBC trapping, their ability to act as natural biotweezers was further investigated. To visualize the focus effect of RBCs on the excitation laser, the short‐pass filter was removed from the front of the CCD camera, allowing the manipulation laser to be observed directly. As shown in Figure 2d, in the absence of RBCs, the laser emitted from the LDMFP formed only a diffuse bright spot at the probe end with no tight focusing observed (Figure 2d1,e1). However, once an RBC was trapped by the LDMFP, the laser beam was re‐focused by the cell on its distal side (Figure 2d2), forming a second localized peak of the optical field within the blood vessel (Figure 2e2), clearly demonstrating the efficient focusing capability of trapped RBCs for the incident laser beam. Furthermore, the full width at half maximum (FWHM) of the beam was experimentally measured by fitting the intensity profile data with a Gaussian function (Figure 2f). Notably, the RBC biotweezers successfully compressed the FWHM of the output optical field from 3.5 to 1.4 µm, i.e., achieving a 2.5‐fold enhancement in optical field localization. This confirms that RBCs can function as natural biotweezers, enabling secondary focusing of the laser beam within blood vessels.

### 2.3 Enhanced Trapping and Manipulation of Microparticles In Vivo

Benefiting from the tight‐focusing capability of RBC biotweezers, micro/nano‐scale targets will experience enhanced optical forces and become stably trapped at the distal end of the RBC. To validate this concept, a series of experimental investigations was conducted. By modulating the six‐axis microstage, the LDMFP was positioned adjacent to the target blood vessel, where polystyrene microparticles (serving as representative drug carriers) were flowing spontaneously within the bloodstream (indicated by deep red arrows in Figure 3a1,b1). Upon illumination with a 980‐nm laser beam (*P* = 80 mW) through the LDMFP, nearby microparticles were not captured by the probe itself but continued their upward movement along the blood flow direction (Figure 3a2,b2). This phenomenon occurs because the optical force scales with the microparticle volume, making stable trapping of smaller particles challenging with the probe alone. However, when an RBC biotweezer was assembled by the LDMFP, stable trapping of microparticles was successfully achieved (Figure 3a3,b3). Subsequent vertical shift of the LDMFP enabled synchronous displacement of both the RBC biotweezers and the target microparticle, facilitating dynamic positioning along the vascular direction (Figure 3c).

**FIGURE 3:**
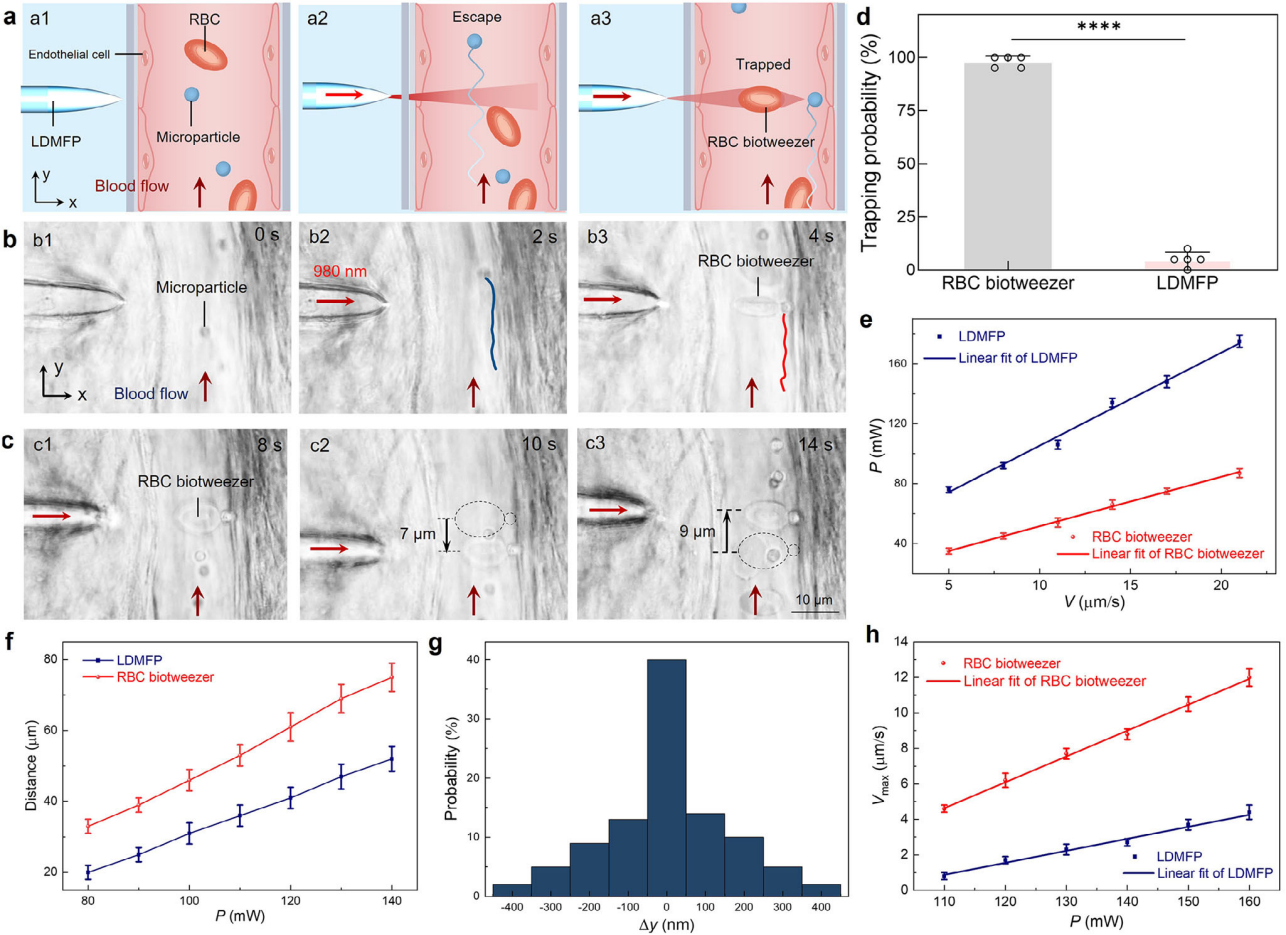
Enhanced microparticle manipulation using RBC biotweezers. (a,b) Schematic illustration (a) and corresponding optical micrographs (b) of enhanced microparticle manipulation using RBC biotweezers. Blue particles represent microparticles, blue gradient lines indicate motion trajectories, and dark red arrows show blood flow direction (*P* = 80 mW, *V*_blood_ = 10 ± 2 µm/s and *d* = 16 µm). (c) Optical micrographs showing dynamic migration of microparticle using RBC biotweezers (*P* = 80 mW, *V*_blood_ = 10 ± 2 µm/s and *d* = 16 µm). (d) Comparison of microparticle trapping probability between RBC biotweezers and LDMFP under identical laser power and flow conditions. Data are mean ± SD from 5 individual zebrafish, with trapping probability determined from 20 independent technical replicates per fish. Statistical comparison was analyzed by two‐tailed *t*‐test with Welch correction, assuming a Gaussian distribution. ^****^ *p*< 0.0001. (e) The required threshold laser power for stable particle trapping as a function of blood flow velocity for both systems. Results are presented as mean ± SD (*n* = 5 individual zebrafish). (f) Maximum particle manipulation distance vs. laser power for RBC biotweezers and LDMFP. Results are presented as mean ± SD (*n* = 5 individual zebrafish with 3 independent replicates per fish). (g) Statistical analysis of particle position offset relative to the focus of RBC biotweezers during migration. (h) Maximum migration velocity of microparticles as a function of laser power for both manipulation systems. Results are presented as mean ± SD (*n* = 6 individual zebrafish with 3 independent replicates per fish).

To quantitatively assess the enhancement effect of RBC biotweezers, the trapping performance of microparticles was systematically compared for two cases (i.e., with and without RBC biotweezers) while under identical laser power (*P* = 80 mW) and blood flow velocity (*V*_blood_ = 10 ± 2 µm/s). The results demonstrated that the RBC biotweezers significantly increased the trapping probability from 5% to 98% when compared to using the LDMFP alone (Figure 3d). Furthermore, under various flow velocities, the minimum laser power required for stable microparticle trapping was markedly reduced after the implementation of RBC biotweezers (Figure 3e), confirming their enhanced capability for manipulating small‐scale targets. Additional investigation into the maximum achievable manipulation distance revealed that the RBC biotweezers substantially increased the effective optical manipulation depth across different laser power levels (Figure 3f). For instance, at a laser power of *P* = 140 mW, the maximum distance for stable particle trapping was extended from 52 to 75 µm.

Moreover, the positional offset (*Δy*), i.e., between the center of the trapped microparticle and the focal point of the RBC biotweezer, was calculated to characterize the stability of migration manipulation along the vessel wall (Figure 3g). The results indicate that the microparticle moved synchronously with the LDMFP throughout the whole migration process, with *Δy* confined within ± 400 nm. Moreover, under identical laser power (*P* = 160 mW) and flow conditions (*V*_blood_ = 10 ± 2 µm/s), the RBC biotweezer enabled a greater counter‐flow migration velocity compared to the LDMFP alone, i.e., 12 µm/s vs. 4.5 µm/s (Figure 3h). This enhanced performance, which stems from the natural light‐focusing characteristics of the RBC biotweezers, might provide a robust platform for biomedical applications requiring precise micromanipulation in vivo.

### 2.4 Immune Cell Activation and Behavioral Regulation

Following the stable trapping and target delivery of microparticles, we further investigated whether the RBC biotweezers could be utilized for the dynamic activation of immune cells. As a key component of the immune system [43], neutrophils can be activated by signaling molecules released from damaged tissues or foreign pathogens [44], subsequently migrating to lesion sites to target and clear damaged cells or invasive pathogens [44, 45]. However, this process primarily relies on their intrinsic biological behavior, making the activation direction and migration speed difficult to control externally [46]. Therefore, the precise activation and directional guidance of neutrophil cells to pathological sites are critical for enhancing their immune capability, such as the active clearance of exotic bacteria. Previous studies have shown that the focused NIR light on immune cells can promote their transition from a quiescent to an activated state and enhance their innate immune response [47, 48, 49]. Consequently, by leveraging the natural focusing properties to enhance the localized optical field, RBC biotweezers are expected to enable efficient neutrophil activation at lower optical power.

To validate this hypothesis, the transgenic zebrafish line of Tg(lyz: dsRed) was used, which exhibited a clear fluorescent labeling and enabled accurate motion tracking (Figure S7). First, the neutrophil cell activation performance was compared for two cases, i.e., with and without the RBC biotweezer system (Figure 4a,b). Initially, the LDMFP was positioned adjacent to target neutrophil cells while illuminated with a 980 nm laser beam at a power of 120 mW (Figure 4a1,a2). After continuous exposure for 40 s, the neutrophils remained in their initial quiescent state without activation (Figure 4a3), indicating that the NIR irradiation from LEMFP alone was insufficient to reach the activation threshold. Subsequently, the LDMFP was used to trap an RBC, assembling the biotweezer system to exploit its tightly focused optical field for neutrophil cell activation (Figure 4b1,b2). Under identical input power (*P* = 120 mW), the enhanced focusing capability of the RBC biotweezer enabled successful cell activation within just 10 s of exposure (Figure 4b3). Pseudopodia formation was observed extending toward the focal point of the RBC biotweezer, which should be attributable to the tightly focused NIR optical field of the RBC biotweezer. Moreover, a sham control group has been performed for the immune cell activation experiments, in which the LDMFP was positioned adjacent to neutrophils without laser illumination (Figure S8a). Under this condition, no immune cell activation was observed for the duration of 120 s, effectively excluding mechanical proximity as a confounding factor. In addition, a flat fiber end‐face was used to deliver non‐focused light, under which the neutrophils cannot be activated (Figure S8b). Together, these control experiments demonstrate that the observed immune cell activation is not due to mechanical proximity or general laser irradiation, but is closely associated with the localized light‐field enhancement induced by the RBC biotweezers. However, it should be noted that the specific neutrophil activation mechanism remains an unresolved question, which might involve photothermal stimulation [47, 50], photochemical effects, or photomechanical stress, and remains a vital and challenging direction for future work.

**FIGURE 4:**
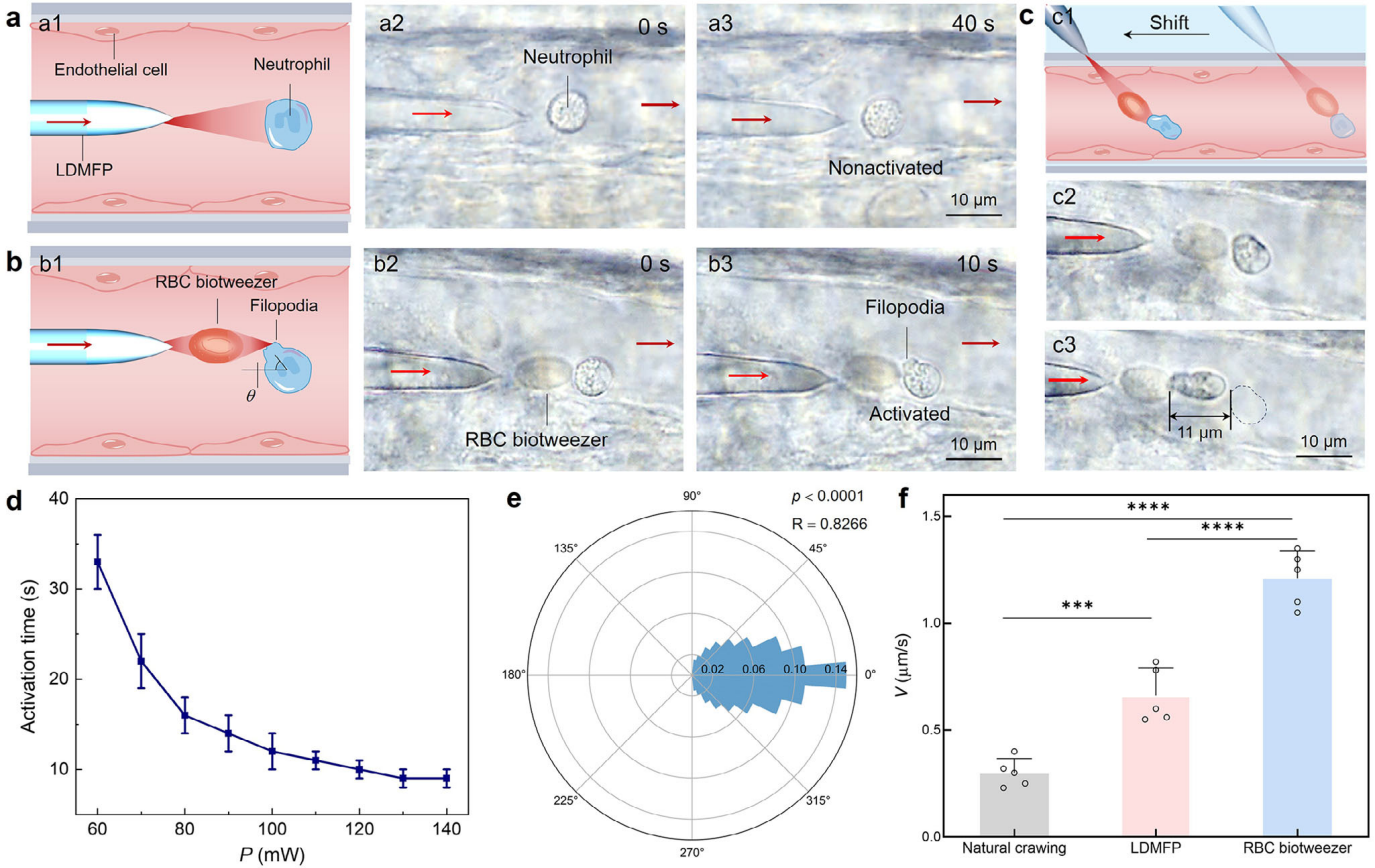
Dynamic activation and controlled migration of immune cells using RBC biotweezers. (a) Schematic diagram (a1) and optical micrographs (a2,a3) showing unrealized neutrophil activation using the LDMFP alone (*P* = 120 mW, *V*_blood_ = 10 ± 1 µm/s and *d* = 3 µm). (b) Schematic illustration (b1) and optical micrographs (b2,b3) of successful neutrophil activation achieved by RBC biotweezers (*P* = 120 mW, *V*_blood_ = 10 ± 1 µm/s and *d* = 3 µm). (c) Schematic representation (c1) and optical micrographs (c2‐c3) of directed neutrophil cell migration by modulating RBC biotweezers (*P* = 120 mW, *V*_blood_ = 10 ± 1 µm/s and *d* = 3 µm). (d) The required activation time as a function of the laser power of the RBC biotweezer. Results are presented as mean ± SD (*n* = 6 individual zebrafish). For each zebrafish, the activation time was measured for 9 individual neutrophil cells, with each one exposed to a unique laser power. (e) Quantitative characterization of the deviation angle between the neutrophil cell activation direction (pseudopodia orientation) and the optical axis of the RBC biotweezers. Data was analyzed using a Rayleigh test. (f) Comparative analysis of migration velocities of activated neutrophil cells under three conditions, including the natural crawling, LDMFP manipulation, and RBC biotweezer control. Data are mean ± SD (*n* = 5 individual zebrafish with 3 independent replicates per fish). Data were analyzed using one‐way ANOVA with Bonferroni correction. ^***^ *p*< 0.001; ^****^ *p*< 0.0001.

Following successful activation, dynamic navigation was attempted using the same RBC biotweezer (Figure 4c). When the biotweezer was translated 11 µm leftward along the blood vessel, the neutrophil cell migrated synchronously while maintaining pseudopodia orientation toward the focus of the RBC biotweezer. This demonstrates the capability to precisely control migration trajectory and direction in real‐time through spatial manipulation of the RBC biotweezer position. Further quantitative investigations were conducted to characterize the above activation and migration process. As the laser power increased from 60 to 140 mW, the activation time decreased correspondingly from 33 to 9 s, demonstrating an enhanced activation efficiency (Figure 4d). Meanwhile, the filopodia mainly grew from the beam focus of the RBC biotweezer and toward the same direction (Figure 4e), confirming that the filopodia originated from the RBC biotweezer rather than a random coincidence. Additionally, the average migration velocity of neutrophil cells in their natural state was approximately 0.3 ± 0.1 µm/s [16], which increased to 0.6 ± 0.2 µm/s under LDMFP manipulation and 1.2 ± 0.2 µm/s under the RBC biotweezer system. Notably, the reported neutrophil migration velocity under LDMFP illumination alone was measured for the neutrophils that were already in an activated state rather than in non‐activated cells. These findings demonstrate that the RBC biotweezer not only enables rapid cell activation but also promotes directional control of cell migration in a remotely controlled manner.

### 2.5 Dynamic Assembly and Functional Characterization of RBC Biotweezer Chain

Building upon the successful assembly of single RBC biotweezers and their enhanced manipulation of micro/nano particles and immune cells, the potential of assembling multiple RBC biotweezers was investigated to extend the manipulation distance further (Figure 5a). Initial experiments validated the capability of the LDMFP to simultaneously manipulate multiple RBCs within living vasculature. As shown in Figure 5b, the LDMFP successfully achieved stable trapping of one (Figure 5b2) and two RBCs (Figure 5b3) at *t* = 1 and 2 s, respectively. The assembled RBC chain maintained controllable migration along the blood vessel (Figure 5b4), confirming the assembly feasibility of multiple RBC biotweezers. Subsequently, a biotweezer chain comprising five RBC biotweezers was constructed (Figure 5c). The laser power required for simultaneously manipulating different numbers of RBCs was quantitatively characterized under two configurations: perpendicular and parallel to the vessel wall. The results indicated that higher laser power was required to manipulate more RBC biotweezers due to inherent tissue absorption and scattering. Crucially, the power requirement for parallel manipulation significantly exceeded that for perpendicular orientation (Figure 5d). However, perpendicular manipulation was limited by vascular width constraints, which might cause RBCs to stack along the *z*‐axis (Figure S9), preventing the construction of long‐distance RBC biotweezer chains. Therefore, parallel alignment along the vascular direction was selected for subsequent array assembly.

**FIGURE 5:**
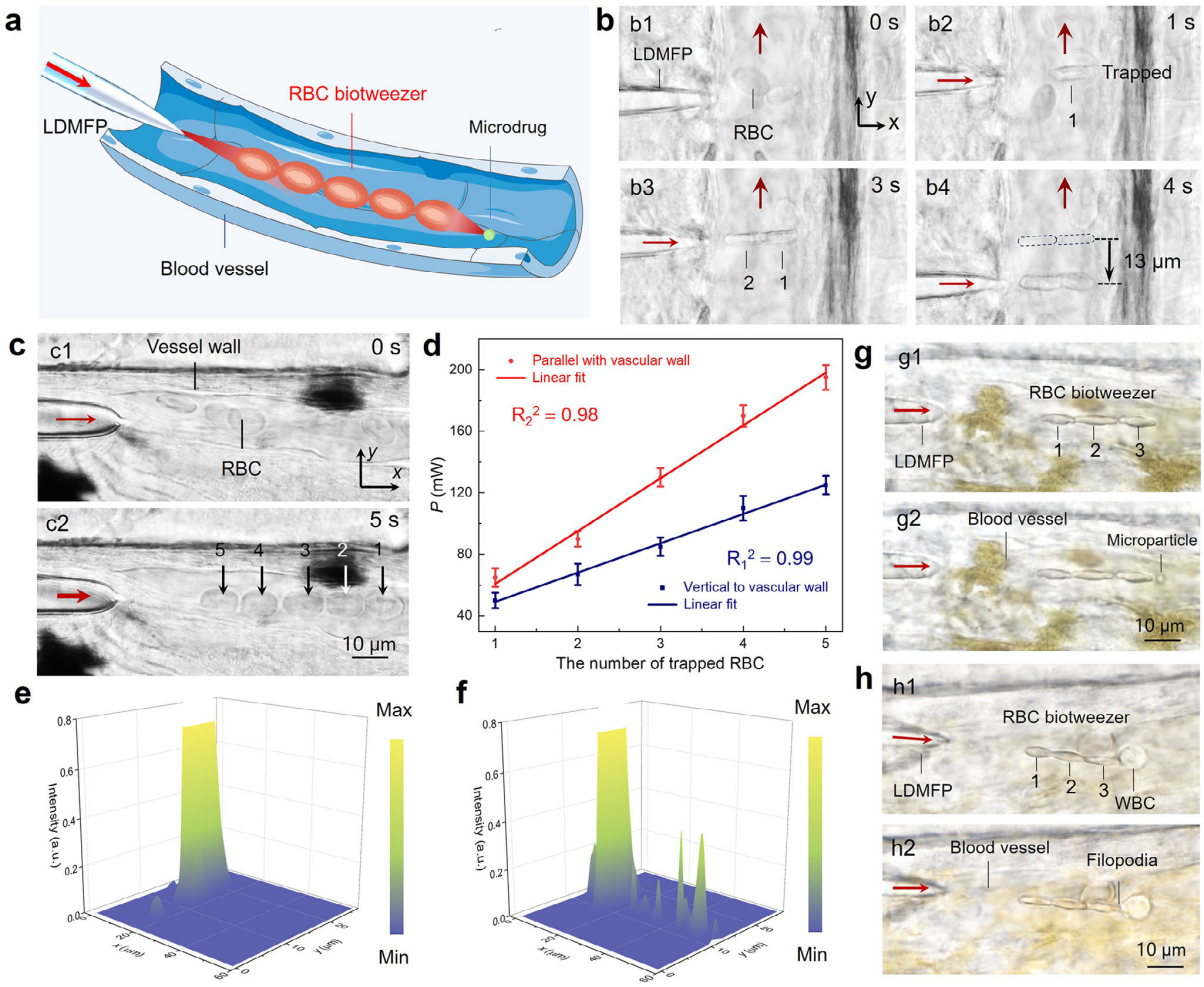
Dynamic assembly and property characterization of multiple RBC biotweezers. (a) Schematic illustration of assembling multiple RBC biotweezers for long‐distance microparticle manipulation. (b) Optical microscopic images showing simultaneous trapping of two RBCs and their dynamic migration using the LDMFP (*P* = 80 mW, *V*_blood_ = 24 ± 5 µm/s, and *d* = 16 µm). (c) Optical microscopic images of the parallel trapping of five RBCs along the vessel wall direction (*P* = 180 mW, *V*_blood_ = 30 ± 5 µm/s and *d* = 23 µm). (d) The required laser power as a function of the number of trapped RBCs. Red and blue curves represent the fiber probe oriented parallel and perpendicular to the vessel wall, respectively. Results are presented as mean ± SD (*n* = 5 individual zebrafish with 3 independent replicates per fish). (e,f) 3D color mapping of the emitted light field without (e) and with (f) RBC biotweezer chain. (g) Long‐distance manipulation of microparticles achieved using a biotweezer chain comprising three RBCs (*P* = 100 mW, *V*_blood_ = 5 ± 1 µm/s and *d* = 27 µm). (h) Dynamic activation of immune cells is accomplished through a three‐RBC biotweezer chain (*P* = 100 mW, *V*_blood_ = 6 ± 1 µm/s and *d* = 21 µm).

Following the simultaneous manipulation of multiple RBC biotweezers, their focusing performance was characterized by analyzing the 3D intensity distribution of the emitted light field with and without a three‐RBC biotweezer array. Compared to the LDMFP alone (Figure 5e), the RBC biotweezers enabled directional laser transmission along the RBC chain, with peak intensity maximized at the chain's terminus (Figure 5f). These findings demonstrate that the RBC chain functions as an optical waveguide for long‐distance directional transmission, achieving strong focusing and localized field enhancement at its distal end. Leveraging this enhanced focusing capability, the desired microparticle manipulation (Figure 5g) and immune cell activation (Figure 5h) were achieved, respectively, with distances of 48 and 40 µm, which confirms that multiple RBC biotweezers can significantly extend the manipulation range beyond the capabilities of standard LDMFP systems alone, providing a potential strategy for flexible manipulation of micro/nano targets in vivo.

### 2.6 Numerical Simulation and Mechanism Analysis

To bridge the gap between experimental observations and theoretical understanding, the underlying processes were numerically simulated and theoretically explained using the finite element method (COMSOL Multiphysics 6.0). As shown in Figure 6a1, unlike conventional optical fiber tweezers, where the focal point of the emitted field is located at the probe tip, the LDMFP employed in this study exhibited long‐distance far‐field focusing with a focal length of 15 µm. This extended focusing capability enabled non‐contact trapping of flowing RBCs within blood vessels (Figure 6a2). Notably, the discrepancy between the simulated focal length (∼15 µm) and the experimentally observed trapping distance (∼30 µm) might arise from the conditions of optical trapping in a dynamic fluidic environment. The simulation identifies the geometric focus (point of maximum intensity) under static and ideal conditions, while the actual intravascular environment includes scattering, absorption, and complex light‐cell interactions. In practice, within flowing blood, stable trapping occurs at the downstream position where the optical gradient force balances the hydrodynamic drag force, i.e., an equilibrium necessarily located beyond the focal point. Thus, the operational trapping distance naturally exceeds the simulated focal length. To calculate the trapping stiffness coefficient *k*_trap_ of the RBC trapped by the LDMFP, the optical gradient force (*F*_o_) acting on the trapped RBC was calculated through integration of the Maxwell stress tensor, which can be expressed as [51, 52]:

(1) $$\langle \vec{F}\rangle =\oint_{A}\langle \bar{T}\rangle \;\cdot n\;dA$$

where *A* represents the closed surface enclosing the RBC, ***n*** is the outward unit normal vector, and $\left\langle \bar{T}\right\rangle$ is the time‐averaged Maxwell stress tensor, which is defined as [23]:

(2) Tij=18πReDiEj∗+BiHj∗−12D·E∗+B·H∗δij

where ***D*** is the electric displacement, ***B*** is the magnetic flux density, ***E*** and ***H*** are the electric and magnetic field intensities, *δ*_ij_ is the Kronecker delta, and *Re* denotes the real part. Figure 6b shows the calculated optical forces along the *y* direction (*F*_y_), which were orientated toward the +*y* or −*y* direction when the RBC was positioned below and above the optical axis of LDMFP, respectively. Meanwhile, the maximum optical force exerted on the target RBC was calculated to be 532 pN/W, enabling stable optical confinement along the optical axis. By calculating the slope at *y* = 0 from the optical force curve, the trapping stiffness coefficient of the LDMFP on RBCs was determined to be *k*_trap_ = 353 pN/µm/W, which is in magnitude agreement with the experimental value of 212 pN/µm/W. To better visualize the trapping capability, the depth of the optical potential well *U_y_* was calculated through integration of *F_y_* along the *y* direction [53]:

(3) $$U_{y}=-\int F_{y}dy$$

**FIGURE 6:**
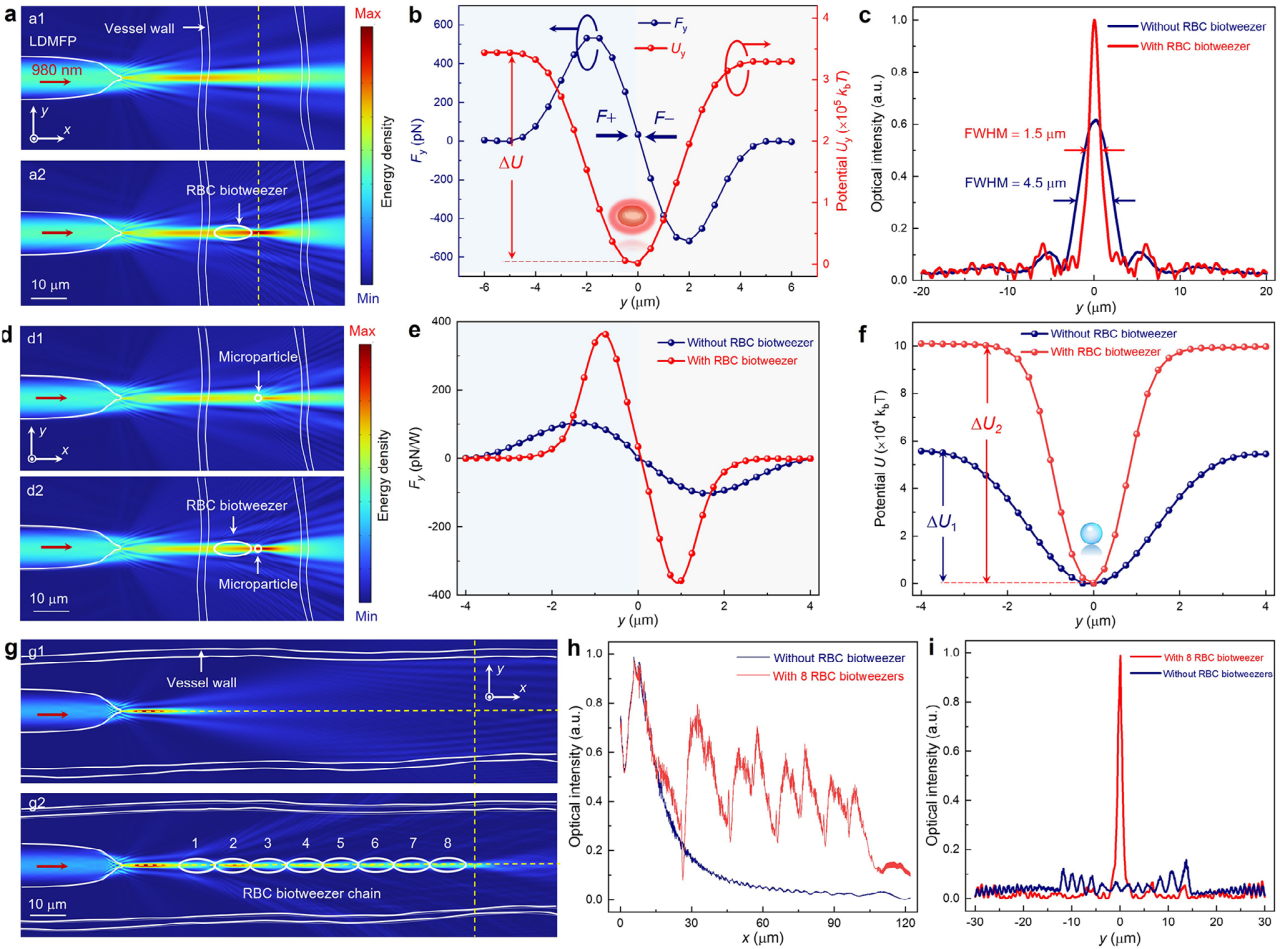
Numerical simulations of optical field distribution and computational analysis of optical force. (a) Simulated energy density distribution of the emitted optical field from the LDMFP (a1) and the RBC biotweezer (a2). (b) The calculated optical force and optical potential well acting on the RBC as a function of the position along the *y*‐direction. (c) Quantitative comparison of the normalized energy density profiles along the *y*‐direction (indicated by the yellow dashed line in a) with and without the RBC biotweezer. (d) Simulated energy density distribution of the emitted optical field during microparticle trapping using the LDMFP (d1) and the RBC biotweezer (d2). (e,f) The calculated optical force (e) and optical potential well (f) acting on the microparticle as functions of the position along the *y*‐position with and without the RBC biotweezer. (g) Simulated energy distribution of the emitted optical field from the LDMFP (g1) and an RBC‐based biotweezer chain (g2). (h,i) Quantitative comparison of the normalized optical filed distribution along the *x*‐direction (i) and *y*‐direction (j) with and without the RBC biotweezer chain, as indicated by the white dashed line and yellow dashed line in g, respectively.

The calculated *U_y_* is shown in Figure 6b with a potential depth of 3.3 × 10^5^ *k*_B_T, which indicates sufficient stability for RBC confinement under the specified conditions [54].

Following the theoretical analysis of stable RBC manipulation, the beam convergence performance of the trapped RBC functioning as a biotweezer was numerically investigated. Compared to the LDMFP alone, the RBC biotweezer effectively concentrated the incident laser beam, forming a distinct focal spot at its distal end. Quantitative analysis of the light field intensity distribution along the *y*‐direction at a fixed distance (i.e., 40 µm) from the probe tip revealed that the RBC biotweezers successfully compressed the FWHM of output optical field from 4.5 to 1.5 µm, achieving a threefold enhancement in optical field localization (Figure 6c). This was consistent with the simultaion‐predicted 2.5‐fold enhancement (Figure 2f), and the minor discrepancies might be attributed to the slight RBC orientation, light scattering in blood or CCD imaging resolution. Based on these, the manipulation performance was systematically explored on the RBC biotweezers (Figure 6d), demonstrating that the secondary tight focusing effect increased the maximum optical force on target particles from 103 to 362 pN/W (Figure 6e), with corresponding optical potential well depth increased from 0.55 × 10^5^ *k*_B_T to 1 × 10^5^ *k*_B_ *T*. These results confirm that the RBC biotweezers establish a 3D potential well within living organism that are capable of enhanced micro particle manipulation, where microparticles become stably trapped at the laser focus unless disturbed by energy exceeding the potential well depth from Brownian motion or blood flow. This comprehensive theoretical framework primarily serve to provide mechanistic plausibility for the observed phenomena (e.g., long‐distance focusing, RBC lensing and waveguiding) and demonstrate order‐of‐magnitude consistency with key experimental trends (e.g., enhancement of trapping stiffness and extension of operational range).

Moreover, numerical simulations were also performed for the assembled RBC biotweezer chain. Compared to a single fiber probe, the presence of RBC biotweezer chain (comprising eight RBC biotweezers) effectively guided the manipulation beam forward along the optical axis (Figure 6g). This configuration significantly extended the optical manipulation distance while further reducing the beam spot size. By comparing the normalized energy density distribution along the *x* direction (as indicated by the white dashed line in Figure 6g), the light field intensity from the bare fiber probe was found to decay rapidly while the intensity profile for the biotweezer chain exhibited a periodically gradual decline (Figure 6h). At a distance of 95 µm from the probe tip, the light intensity emitted from the single LDMFP was diminished to zero. However, the intensity remained at 50% of its maximum value in presence of RBC biotweezer, followed by a sharp decline nearby. This characteristic profile suggests the potential formation of a strong optical gradient force along the *x*‐direction for enhanced microparticle manipulation. Furthermore, the emitted light field intensity distribution along the *y*‐direction (indicated by the yellow dashed line in Figure 6g) was also characterized. In the presence of RBC biotweezer chain, a distinct intensity peak emerged near *y* = 0 µm, indicating the potential for stable confinement of target microparticles along the optical axis and enabling their enhanced trapping.

Furthermore, the influence of the number and arrangement of RBCs on the light field propagation and focusing effect was also analyzed. Notably, the laser beam is effectively guided along the optical axis as the RBC number increases, thus enabling a controllable extension of the operational range of the optical potential well. This provides a quantitative explanation for the extended manipulation distances observed experimentally with multiple biotweezer arrays (Figure S10). Meanwhile, a non‐ideal, physiologically relevant scenario was modeled where the flow may cause the long axis of RBC biotweezers to deviate from the optical axis (Figure S11). The propagating beam undergoes significant deflection in the case of a misaligned cell arrangement, which leads to compromised beam transmission along the remainder of the chain and results in degraded focusing performance at its terminus. This analysis reveals the importance of alignment for efficient waveguiding and predicts the performance tolerance of the system under realistic conditions in vivo. These simulation results demonstrate the dynamic assembly and manipulation mechanisms of RBC biotweezers using the LDMFP, which establish a solid foundation for understanding the physical principles governing the performance of biological optical manipulation platforms in complex physiological environments.

## 3 Discussion

In this study, we have demonstrated a novel RBC biotweezer by integrating engineered fiber optics with endogenous cellular components. The developed LDMFP enables non‐contact capture and assembly of RBCs within living vasculature, while the captured RBCs themselves function as natural biotweezers to create secondary optical traps at extended working distances. The experimental results in zebrafish models validate several key advancements, including the stable capture and manipulation of individual RBCs, the enhanced trapping of microparticles through RBC biotweezer focusing, and the controlled activation and directional migration of immune cells. Furthermore, multiple RBCs can be assembled into functional biotweezer arrays, acting as biological waveguides to propagate optical fields into deeper tissue environments. Finite‐element simulations provide a quantitative interpretation of the observed phenomena, revealing the underlying mechanisms of extended focusing and enhanced optical gradient forces.

To determine the “stable working time” of the proposed RBC biotweezer, individual RBCs were trapped at the maximum laser power used in this work (i.e., *P* = 240 mW). Notably, the RBC biotweezer remains in its normal shape without any discernible morphological alterations, even under continuous laser irradiation for 300 s (Figure S12a). It might be attributed to the low absorption of RBCs on the 980 nm light beam [55, 56], and meanwhile, the absorption‐induced heat could be effectively conducted and diffused due to the high thermal conductance of cells and the fast blood flow in vessels. However, the thermal equilibrium mechanism might be broken under the higher laser power (*P* = 400 mW), and meanwhile, the absorption‐induced heat will gradually accumulate until a significant rupture is observed for the RBC biotweezer with a working time of 15 s (Figure S12b). Besides, there was no immediate tissue damage (e.g., bleeding, visible lesion formation, or dramatic change in local vasculature) during and immediately after all manipulation sessions, suggesting the proposed RBC biotweezers are anticipated to be biocompatible given the use of a single‐cell‐diameter fiber probe and NIR light with relatively deep tissue penetration. However, its full biocompatibility and invasiveness require future systematic validation in vivo, including histological analysis and markers of inflammation.

Moreover, the influence was also explored for the physiological factors, such as blood flow rate and vascular diameter on the assembly and manipulation effect of RBC biotweezers. With the increase in blood flow velocity, a correspondingly higher laser power is required to achieve successful cell trapping and assembly (Figure 2b). However, elevated flow rates substantially compromise trapping stability, particularly for multi‐RBC assemblies, which are more susceptible to disruption due to increased drag forces. To mitigate this effect, blood flow conditions were regulated by carefully adjusting the dosage and duration of anesthesia, thereby maintaining the flow velocity within a desired range of 10–50 µm/s. In addition, the vascular diameter will also determine the assembly stability and operational feasibility of the RBC biotweezer. Notably, the vessels with excessively small diameters (i.e., < 10 µm) impose additional confinement and wall‐induced resistance, while the vessels with larger diameters (i.e., > 50 µm) typically exhibit higher cell density and less controllable flow conditions, both of which increase the difficulty of achieving stable trapping, especially for multi‐cell configurations. Thus, vessels with diameters in the range of 10–30 µm were preferentially selected in this study.

The initial simulations employed a 2D model for computational efficiency, particularly for extended RBC chains. While this simplification captures the fundamental physics (e.g., long‐distance focusing and qualitative lensing behavior) and supports the key qualitative conclusions from experiment, it inherently simplifies the true 3D biconcave geometry. This likely leads to a quantitative underestimation of optical forces and potential well depths, as 3D curvature would enhance light confinement. Therefore, future high‐fidelity 3D simulations, though computationally intensive, would be valuable for obtaining precise quantitative predictions. Moreover, a dedicated sensitivity analysis was performed to verify the impact of the RBC refractive index, which was the most critical parameter governing the light‐focusing (lensing) efficiency and thus the core performance of the RBC biotweezer. The analysis was conducted across the physiologically relevant range for RBCs, i.e., from 1.38 to 1.42 [32]. Notably, an increase in the refractive index systematically shifts the focal point closer to the RBCs' distal end (Figure S13). Concurrently, the FWHM of the focused spot becomes narrower, and its peak intensity increases, confirming a direct relationship between the RBCs intrinsic optical property and the biotweezers' focusing performance.

Notably, conventional high‐numerical‐aperture (high‐NA) objective‐based optical tweezers are well established and capable of generating strong trapping stiffness [57, 58], while they typically rely on bulky free‐space optical setups, involve high system cost, and require stringent alignment conditions. Benefiting from the miniaturized geometry and flexible integration, fiber‐based optical tweezers enable desired insertion into deep tissue and facilitate precise optical manipulation within otherwise inaccessible biological environments. However, the current mainstream commercial fiber optical tweezers exhibited a limited capture stiffness [59, 60] due to the unimproved focusing capability. In contrast, the proposed LDMFP inherits the intrinsic advantages of fiber‐based platforms, while the incorporation of RBC biotweezers further enhances trapping stiffness (see Table S1 for the detailed comparison). Meanwhile, unlike conventional methods that require exogenous implants or chemical modifications, the proposed RBC biotweezer leverages naturally occurring cellular structures, thereby minimizing immune responses and biocompatibility concerns while maintaining full biological functionality [27]. Furthermore, the hierarchical optical design achieves improved manipulation depths within living organisms. By combining the long‐range focusing capability of the specialized fiber probe with the light‐guiding properties of RBC biotweezers, the established multi‐stage optical system represents a step forward in addressing the depth limitations plaguing conventional optical tweezers [61, 62]. Moreover, its adaptability to different biological scenarios suggests potential applications beyond therapeutic interventions, such as fundamental research in cell biology and biophysics. Most importantly, this research establishes an alternative concept where intravital cells become active components of optical systems, rather than merely passive manipulation targets, which might inspire the development of bio‐hybrid technologies that combine engineering precision with natural biological systems.

While this proof‐of‐concept study demonstrates promising capabilities, several challenges require further investigation. The long‐term stability of RBC biotweezers under continuous optical exposure needs systematic evaluation to determine optimal operational parameters and duration limits. Additionally, comprehensive studies on the effects of optical manipulation on cellular viability, membrane integrity, and physiological function are essential for clinical translation [63]. Moreover, adaptation to diverse tissue environments will require careful optimization, given the substantial variations in optical properties, mechanical characteristics, and biological context across different organs. Integrating real‐time imaging with closed‐loop feedback control represents an important direction to improve manipulation precision and robustness. In this regard, the development of intelligent control strategies capable of dynamically responding to biological feedback and environmental perturbations could significantly enhance applicability in complex physiological settings [64]. In addition, combining this approach with complementary manipulation modalities may provide synergistic advantages for more demanding scenarios.

Notably, the optical transparency of the zebrafish model is the fundamental enabler for the real‐time visualization and optical manipulation demonstrated in this study. However, translating this concept to human vasculature presents significant challenges due to fundamentally different optical and physiological conditions, including increased tissue scattering and absorption, greater vessel depth, and more complex hemodynamic environments. Overcoming these challenges will require substantial methodological re‐engineering rather than incremental optimization, such as employing longer‐wavelength excitation in the near‐infrared II (NIR‐II) window, integrating adaptive optics and wavefront‐shaping technologies, miniaturizing and optimizing fiber probe geometries for intravascular or endoscopic deployment, and incorporating complementary multimodal guidance strategies, which might highlight the opportunities and challenges in translating RBC biotweezers toward more complex and clinically relevant settings.

## 4 Conclusion

In conclusion, this work presents a new biohybrid strategy for in vivo optical manipulation that bridges the gap between optical engineering and cellular biology. By leveraging the innate properties of RBCs as biological microlenses and waveguides, we have developed a natural biotweezer that enables precise micro‐ and nano‐scale operations in complex vasculature environments. This innovative strategy enables the dynamic assembly of both single RBC biotweezers and multi‐cellular biotweezer arrays, which demonstrate successful biomedical applications in enhanced trapping and spatial repositioning of model drug carrier particles as well as optically guided neutrophil behavior in zebrafish models. By combining the biocompatibility of cellular components with the precision of optical manipulation technologies, the proposed RBC biotweezer might provide multifunctional intervention in living organisms, offering promising opportunities for targeted therapeutic delivery, behavior modulation of immune cells and fundamental biological research.

## 5 Experimental Section

### 5.1 Experiment Setup

The fabricated LDMFP was connected to a 980 nm near‐infrared laser source with its probe tip extending approximately 1 cm beyond the glass capillary tube. After that, the LDMFP was mounted on a six‐axis microstage, enabling 3D positional control with a resolution of 500 nm. An adult zebrafish was positioned with its tail oriented toward the fiber probe. The microscope focus was adjusted to clearly visualize blood vessels in the zebrafish tail. By modulating the six‐axis microstage, the fiber probe was positioned directly above the target vessel, followed by fine‐tuning of the *z*‐axis and tilt controls until both the probe tip and flowing red blood cells were simultaneously in clear focus. The laser was then activated at an appropriate power level, and the probe was finely adjusted to achieve stable trapping and 3D manipulation of target RBCs. Illumination was provided by a white LED source, with the light condensed through a condenser lens onto the zebrafish tail vasculature. The experimental images were collected by a 60× water‐immersion objective, passed through a short‐pass filter to block the 980 nm manipulation laser, and finally transmitted via a CCD camera to a computer for real‐time observation, image acquisition and video recording.

### 5.2 Fabrication of the LDMFP

The used LDMFP was fabricated from a commercial single‐mode fiber (connector type: FC/PC, core diameter: 9 µm, cladding diameter: 125 µm; Corning Inc.) by using a flame‐heating method. Initially, the fiber was inserted into a capillary tube (inner diameter: ∼0.9 mm, wall thickness: ∼0.1 mm, length: ∼120 mm) to provide mechanical support and prevent bending or damage during processing. The buffer layer and polymer coating were removed from the fiber end using fiber strippers, obtaining a ∼3 cm long bare fiber segment with a diameter of 125 µm. During the pulling process, the bare fiber was heated approximately 3 mm above the outer flame of an alcohol lamp for about 10 s until the local region reached a near‐melting state. Axial stretching was subsequently performed with an initial speed of 0.4 ± 0.1 mm/s, gradually reducing the fiber diameter from 125 to 9.4 µm over a 2 mm length. The pulling speed was then increased to about 10 mm/s, further tapering the diameter from 9.4 to 8 µm over a 70 µm length. Finally, the speed was accelerated to 20 mm/s, leading to a breakage after achieving a pulled length of 22–30 µm under controlled conditions. The fabricated fiber probe features a smooth and parabolic tip with excellent geometric symmetry and optical transmission properties, fulfilling requirements for subsequent long‐distance optical trapping experiments. Notably, more than 10 probes could be fabricated in the same batch, and the flame‐heating technique used yields >90% geometric consistency across different probes (Figure S14), confirming high reproducibility to minimize inter‐probe variability.

### 5.3 Sample Preparation of Zebrafish

Adult zebrafish (150 days old) were purchased from the Nanjing Eze‐Rinka Biotechnology Co., Ltd. (Nanjing, China) and were then fed with live brine shrimps and cultured with a 14 h light/10 h dark cycle at 28.5°C. The adult zebrafish of the AB strain were utilized for the primary biotweezer investigations, while the Tg(lyz:dsRed) transgenic line was introduced for the experiments requiring neutrophil visualization. To control for sex as a biological variable, zebrafish of both sexes were used in approximately equal numbers, with their body weight ranging from 0.25 to 0.4 g. To maintain physiological viability during experiments, the zebrafish were systemically anesthetized prior to experimentation using a tricaine methanesulfonate (MS‐222) solution at a concentration of 0.3 mg/mL. The anesthesia was considered effective when the fish exhibited no response to external mechanical stimuli, such as gentle tapping of the experimental platform. Subsequently, the anesthetized zebrafish was placed on a standard microscope slide (dimensions: 25 mm × 50 mm) and positioned on the translation stage to achieve high‐precision localization in the *x*–*y* plane. To maintain a stable anesthetic state throughout the experiment, approximately 1 mL of the tricaine solution was periodically replenished around the zebrafish to prevent awakening. All experimental procedures were strictly performed in accordance with the ethical standards and approved by the Laboratory Animal Ethics Committee of Guangzhou University (No. [2025]008).

### 5.4 Blood Flow Measurement

The velocity of blood flow was determined by tracking the displacement of individual red blood cells using ImageJ software. Specifically, RBC positions were tracked frame‐by‐frame from recorded videos, and the flow velocity was calculated as the displacement divided by the corresponding time interval based on the known frame rate. To ensure accuracy and reproducibility, multiple RBCs were tracked for each experimental condition (*n* = 6), and the associated measurement uncertainty has been supplemented with the flow velocity reported as mean ± standard deviation (SD).

### 5.5 Numerical Simulation

The numerical simulation was performed using a 2D finite element method with the radio frequency‐domain module (COMSOL software) and scattering boundary conditions. The incident laser injected into LDMFP was set as an unpolarized Gaussian beam with a wavelength of 980 nm. In the simulation, independent meshes were constructed for the LDMFP, vessel wall, plasma, RBC and PS microparticle, with element sizes of 50, 80, 100, 40, and 20 nm, respectively. To ensure computational accuracy and convergence, a refined mesh was applied to regions with significant light field distribution. Meanwhile, the refractive indices of the LDMFP, vessel wall, plasma, RBC, and PS microparticle were set as 1.44 [65], 1.39 [66], 1.35 [67], 1.40 [32], and 1.58 [68], respectively.

### 5.6 Statistical Analysis

The statistical analysis was performed using GraphPad Prism (v8.0, GraphPad Software, Inc.). Results are presented as mean ± standard deviation (SD). Comparison between two groups was performed by two‐tailed *t*‐test with Welch correction, assuming a Gaussian distribution. Comparisons among multiple groups were performed by one‐way ANOVA with Bonferroni correction. A *p*< 0.05 was considered to be statistically significant.

## Author Contributions

**Hao Pang**: validation, methodology, software. **Tong Yang**: methodology, data curation, investigation, writing – original draft. **Kaize Cai**: validation, formal analysis. **Mei Yuan**: validation, formal analysis. **Xiaoshuai Liu**: project administration, conceptualization, investigation, writing – review and editing, supervision. **Bingzhi Zhang**: writing – review and editing, supervision. **Wei Chen**: validation, visualization. **Xinyu Ren**: investigation, validation, formal analysis. **Zufang Lin**: conceptualization, writing – review and editing, funding acquisition. **Dalin Ma**: formal analysis, resources.

## Conflicts of Interest

The authors declare no conflicts of interest.

## Supporting information




**Supporting File**: advs76794‐sup‐0001‐SuppMat.docx.

## Data Availability

The data that support the findings of this study are available from the corresponding author upon reasonable request.
