# Supplementary material for: Red Blood Cells as Endogenous Biotweezers for Optical Micromanipulation In Vivo
Source: Adv Sci (Weinh). 2026 Jul 23:e76794. Online ahead of print. doi: 10.1002/advs.76794 (PMC13395187; doi:10.1002/advs.76794)
Supplement: Supplementary file 1 — Supporting File: advs76794‐sup‐0001‐SuppMat.docx. [file ADVS-9999-e76794-s001.docx]

Supplementary Materials

Red blood cells as endogenous biotweezers for optical micromanipulation in vivo

Tong Yang^1^, Xinyu Ren ^1^, Dalin Ma^1^, Hao Pang^1^, Wei Chen^1^, Kaize Cai^1^, Mei Yuan^1^, Bingzhi Zhang^1*^, Zufang Lin^2*^, Xiaoshuai Liu^1*^

^1^Department of Optoelectronic Engineering, School of Physics and Materials Science, Guangzhou University, Guangzhou, Guangdong, China

^2^ College of Artificial Intelligence and Low-Altitude Technology, South China Agricultural University, Guangzhou, Guangdong, China.

**E-mail**: zhang_bzh@gzhu.edu.cn (B. Z.), linzf@scau.edu.cn (Z. L.) or lxshuai@gzhu.edu.cn (X. L.)

**Table of Contents**

**Figure S1**. Quantitative characterization of size distributions for the zebrafish RBCs.

**Figure S2**. Schematic illustration of experiment setup.

**Figure S3**. Dynamic trapping and controlled release of RBCs in vivo.

**Figure S4**. Flexible navigation of the trapped RBC along the z axis.

**Figure S5**. Biocompatibility characterization of the LDMFP on RBC.

**Figure S6**. Experimental calculation of the trapping stiffness.

**Figure S7**. Fluorescent imaging of neutrophil cell within the blood vessel.

**Figure S8**. Characterization of neutrophil activation on mechanical proximity and general laser irradiation.

**Figure S9**. Simultaneous manipulation of four RBCs with LDMFP perpendicular to vessel wall.

**Figure S10**. Characterization of the RBC number on the light field propagation and focusing.

**Figure S11**. Characterization of the RBC arrangement on the light field propagation and focusing.

**Figure S12**. Characterization of the working time of RBC biotweezer.

**Figure S13**. Parameter sensitivity analysis for the refractive index on optical trapping performance.

**Figure S14**. Fabrication reproducibility characterization of the LDMFP.

**Table 1**. Key characteristics comparison between different optical tweezers.

1. **Quantitative characterization of size distributions for the zebrafish RBCs.**

**
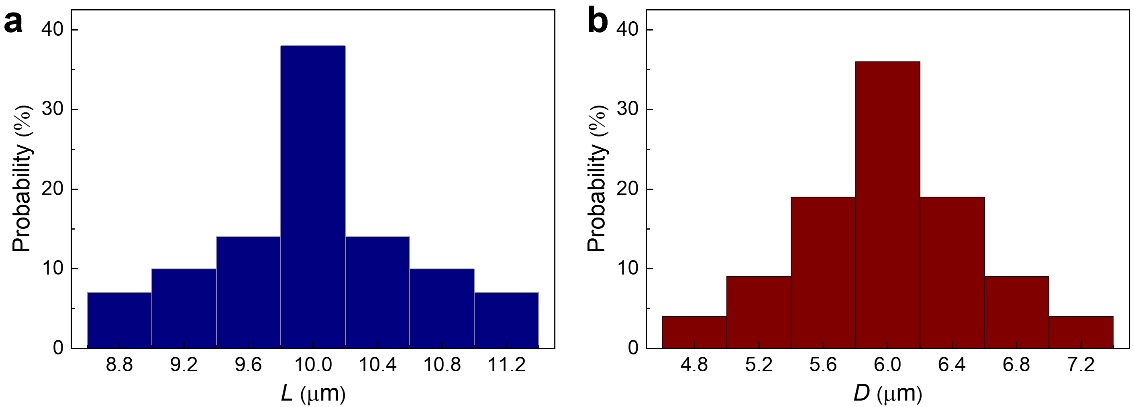
**

**Figure S1**. The calculated probability distribution for the long axis (b1) and short axis (b2) of zebrafish RBCs in blood vessels.

1. **Schematic illustration of experiment setup.**


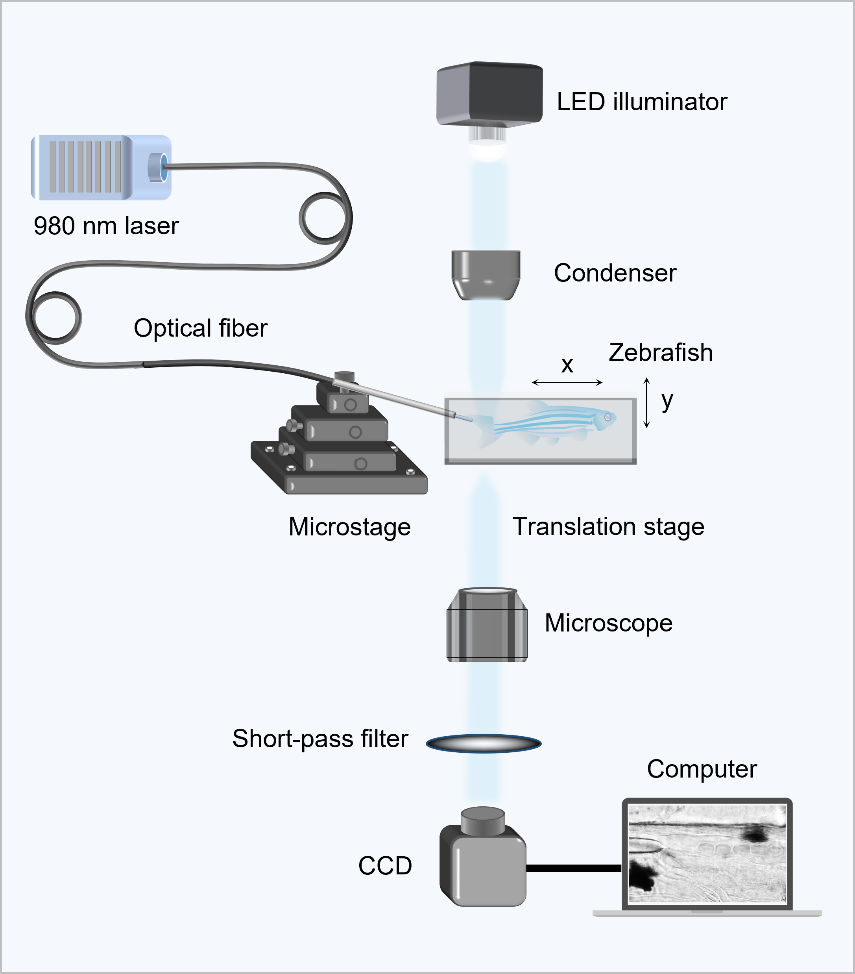


**Figure S2**. Schematic illustration of experiment setup.

1. **Dynamic trapping and controlled release of RBCs in vivo.**


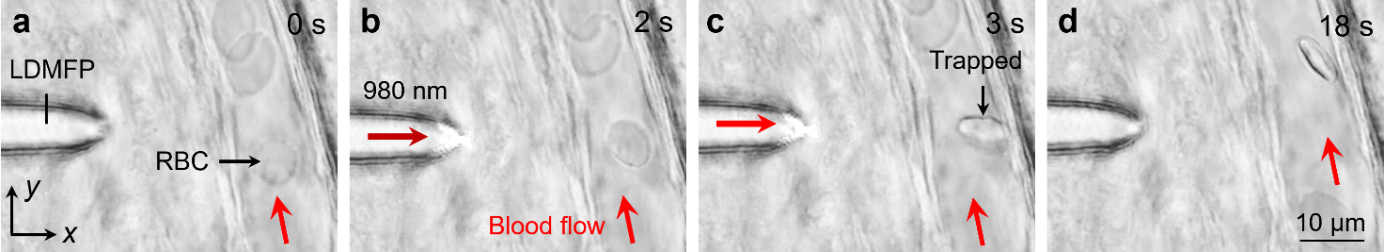


**Figure S3**. Optical micrographs for dynamic trapping and controlled release of RBCs in vivo (*P* = 90 mW, *V*_blood_ = 5 ± 1 μm/s and *d* = 24 μm).

1. **Flexible navigation of the trapped RBC along the z axis.**


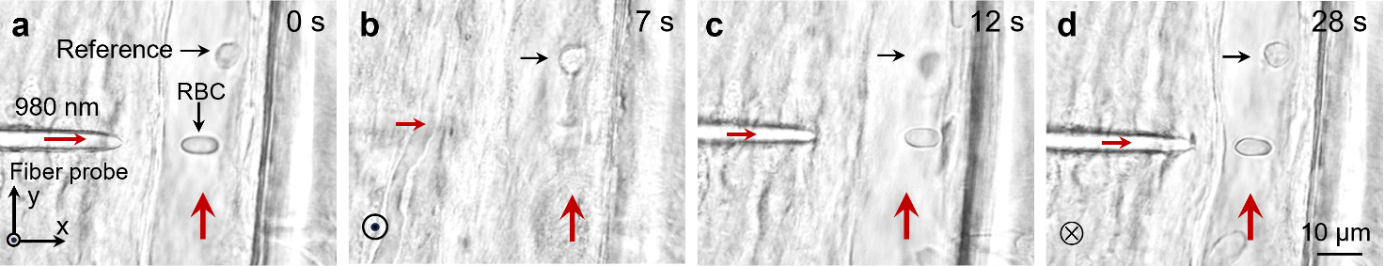


**Figure S4**. Optical micrographs for shifting the trapped RBC along the z axis (*P* = 50 mW, *V*_blood_ = 5 ± 1 μm/s and *d* = 16 μm).

1. **Biocompatibility characterization of the LDMFP on RBC.**


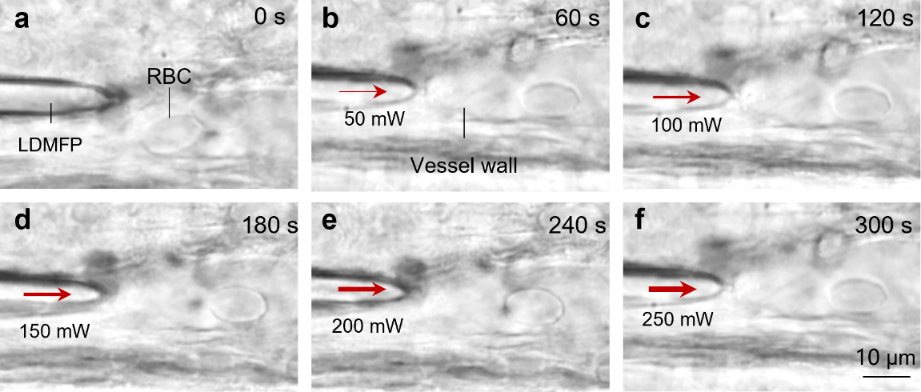


**Figure S5**. Optical micrographs for trapping RBCs under different laser powers (*V*_blood_ = 14 ± 2 μm/s and *d* = 21 μm).

1. **Experimental calculation of the trapping stiffness.**

The optical trapping stiffness (*k*_trap_) has been experimentally validated using the power spectral density (PSD) analysis of positional fluctuations, which is well-suited for in vivo conditions where applying a controlled drag force for escape measurements is challenging. Firstly, the displacement of the trapped RBCs was recorded (*P* = 200 mW) through a quadrat photodiode at high frequency (1000 Hz), which was further treated to generate the power spectrum density (PSD). After that, the PSD was fitted with the Lorentzian model for an overdamped harmonic oscillator, yielding the experimental corner frequency *f*_c_ of 19.84 Hz (**Figure S6**), from which the trapping stiffness *k*_trap_ can be calculated by: *k*_trap_ = 2π*βf*_c_, where *β* =6π*ηr* = 1.70 × 10^−7^ N/m⋅s is the Stokes drag coefficient. Based on these, the optical trapping stiffness was measured to be *k* = 212 pN/μm/W.


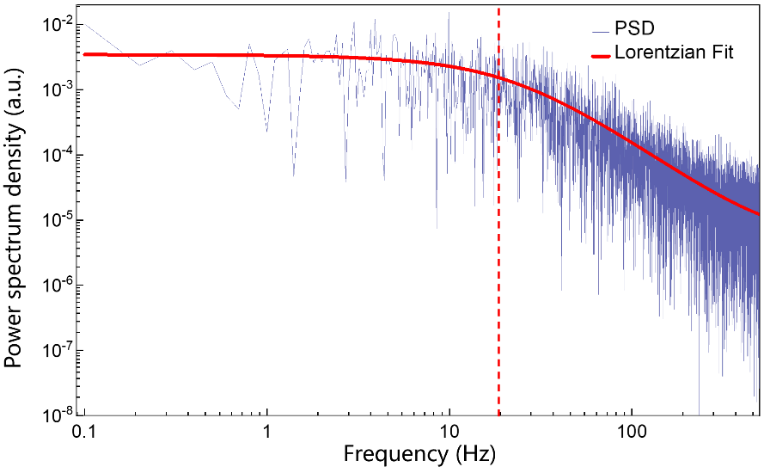


**Figure S6**. The calculated power spectral density during the RBC trapping in vivo and the fitted curve (red) to the Lorentzian model with a corner frequency of *f*_c_ = 19.84 Hz.

1. **Fluorescent imaging of neutrophil cell within the blood vessel.**


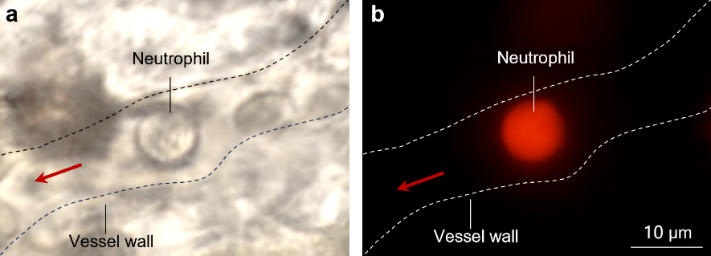


**Figure S7**. Bright-field (**a**) and corresponding fluorescence image (**b**) for the neutrophil cell in blood flow (*V*_blood_ = 40 ±5 μm/s).

1. **Characterization of neutrophil activation on mechanical proximity and general laser irradiation.**


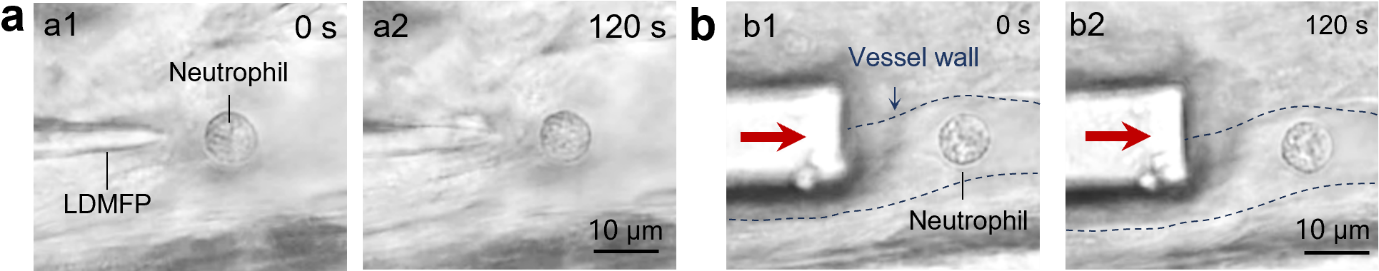


**Figure S8**. (a) Optical micrographs for positioning the LDMFP adjacent to neutrophils without laser illumination (*V*_blood_ = 5 ± 1 μm/s and *d* = 5 μm). (b) Optical micrographs for activating neutrophils with non-focused light from a flat-end fiber probe (*P* = 120 mW, *V*_blood_ = 30 ± 5 μm/s and *d* = 15 μm).

1. **Simultaneous manipulation of four RBCs with LDMFP perpendicular to vessel wall.**


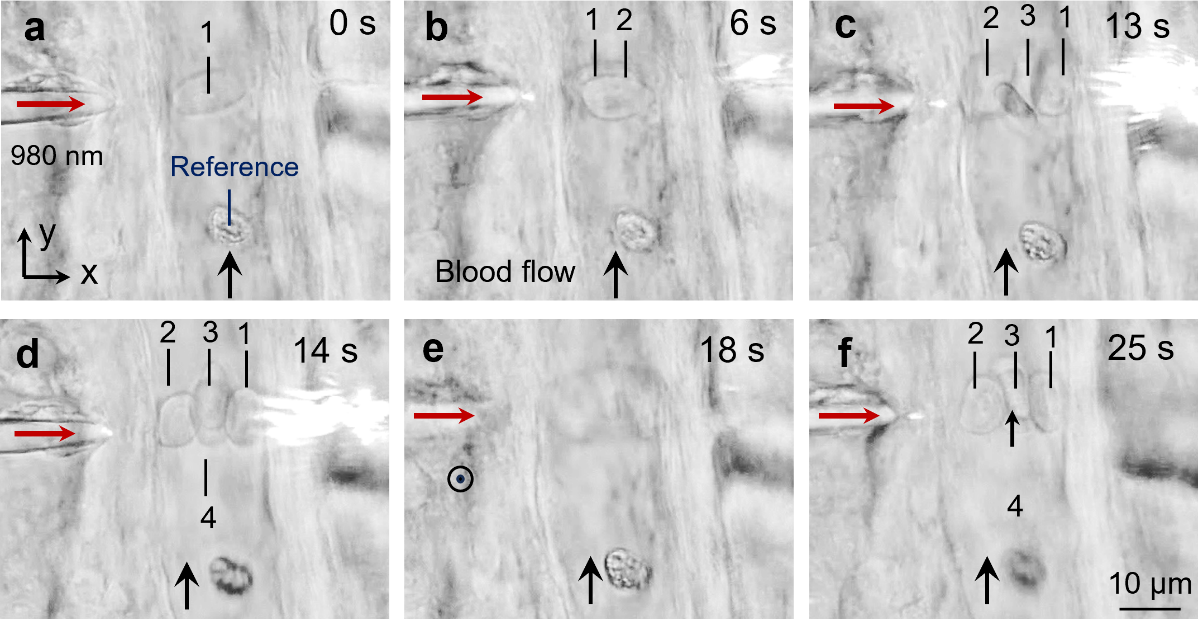


**Figure S9**. Optical micrographs for simultaneous manipulation of four RBCs with the LDMFP perpendicular to the vessel wall (*P* = 150 mW, *V*_blood_ = 8 ± 2 μm/s and *d* = 10 μm).

1. **Characterization of the RBC number on the light field propagation and focusing.**


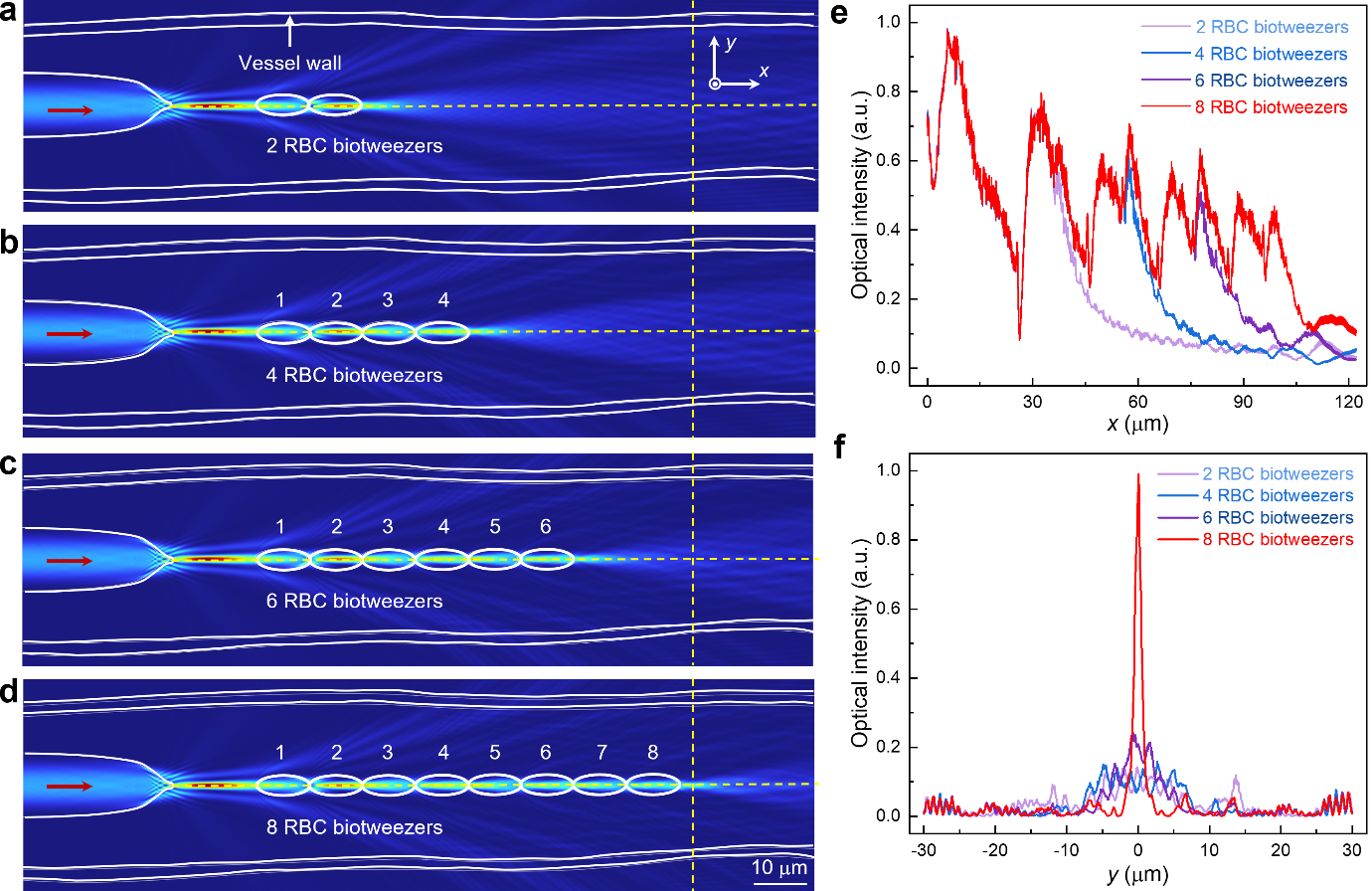


**Figure S10.** (**a-d**) Simulated energy density distribution of the RBC biotweezer chain with different cell numbers. (**e,f**) Quantitative comparison of the normalized energy density profiles along the *x*-direction (**e**) and *y*-direction (f), as indicated by the white and yellow dashed line in a-d, respectively.

1. **Characterization of the RBC arrangement on the light field propagation and focusing.**


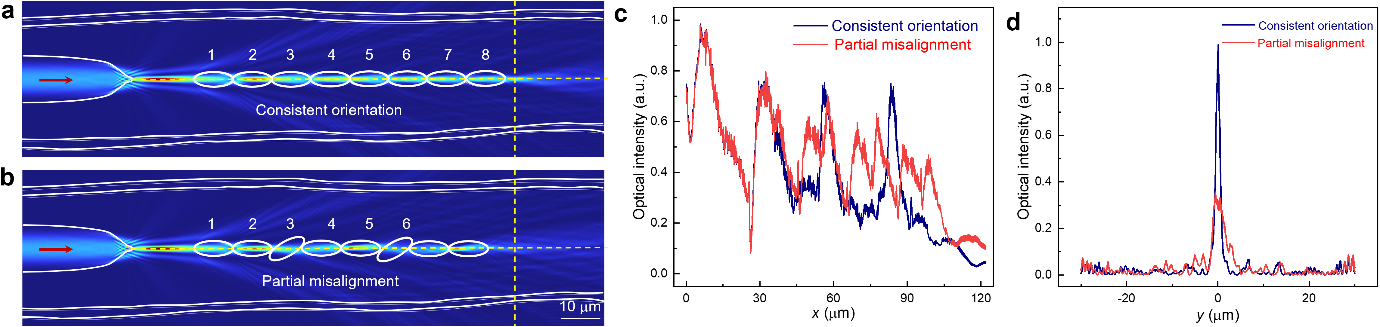


**Figure S11.** (**a, b**) Simulated energy density distribution of the RBC biotweezer chain with consistent orientation and partial titlting (**b**). Quantitative comparison of the normalized energy density profiles along the *x*-direction (**c**) and *y*-direction (**d**), as indicated by the white and yellow dashed line in **a** and **b**, respectively.

1. **Characterization of the working time of RBC biotweezer.**


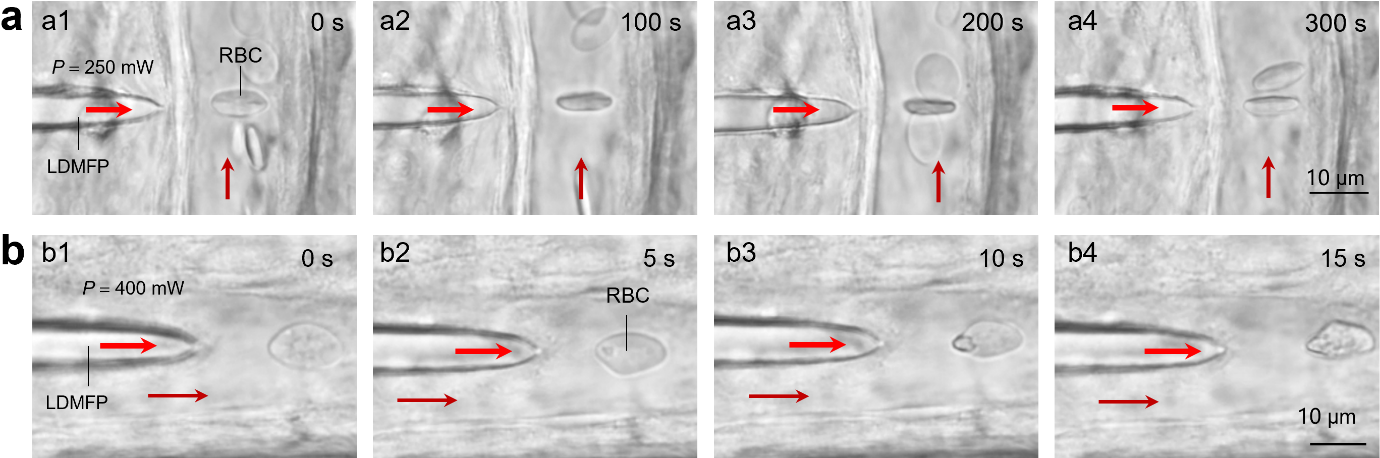


**Figure S12**. (**a**) Optical micrographs for optically trapping RBC at the laser power of 250 mW (*V*_blood_ = 20 ± 2 μm/s and *d* = 11 μm). (**b**) Optical micrographs for optically trapping RBC at the laser power of 400 mW (*V*_blood_ = 20 ± 2 μm/s and *d* = 12 μm).

1. **Parameter sensitivity analysis for the refractive index on optical trapping performance**


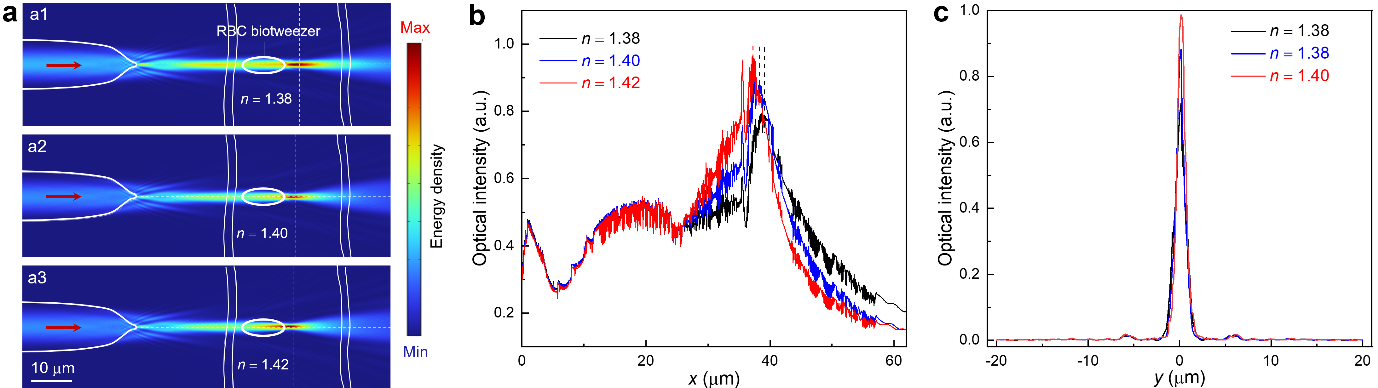


**Figure S13**. (a) Simulated energy density distribution of the RBC biotweezer across the refractive index range of 1.38~1.40. (b,c) Quantitative comparison of the normalized energy density profiles along the x-direction (b) and y-direction (c), as indicated by the white and yellow dashed line in a1-a3, respectively.

1. **Fabrication reproducibility characterization of the LDMFP**


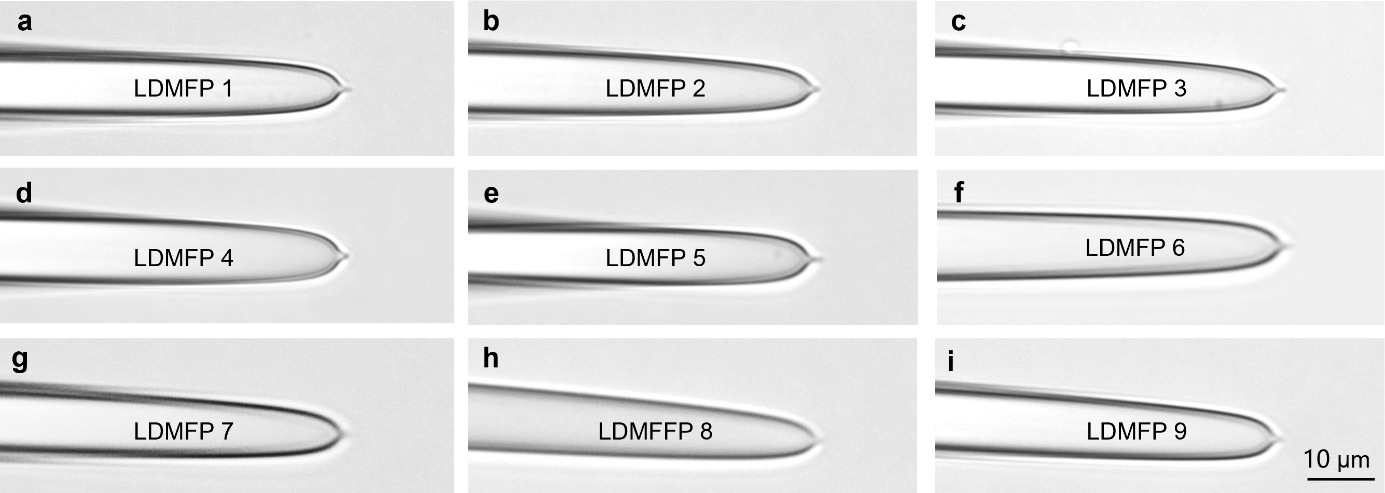


**Figure S14**. Optical micrographs of the fabricated LDMFP with similar shapes.

**Table 1**. Key characteristics comparison between different optical tweezers

| System Type | Capture Stiffness (pN/μm/W) | Key Characteristics | Reference |
| --- | --- | --- | --- |
| Traditional high-NA objective optical tweezers | 260 | High performance, large size, high cost | Opt. Express **2008**, 16, 20987;  Sci. Rep. **2022**, 12., 10229 |
| Commercial fiber optical tweezers | 10-40 | Easy integration, compact, relatively low stability, short working distance | Sci. Rep. **2020**, 10, 20099;  Nanoscale **2022**, 14, 6941 |
| RBC Biotweezers | 353 | Easy implementation, large manipulation distance, and high stiffness | This work |
